# Astrocyte regulation of behavioral outputs: the versatile roles of calcium

**DOI:** 10.3389/fncel.2025.1606265

**Published:** 2025-05-15

**Authors:** Gillian Imrie, Isabella Farhy-Tselnicker

**Affiliations:** ^1^Department of Biology, Texas A&M University, College Station, TX, United States; ^2^Texas A&M Institute for Neuroscience (TAMIN), Texas A&M University, College Station, TX, United States; ^3^Center for Biological Clocks Research, Texas A&M University, College Station, TX, United States

**Keywords:** astrocyte, neuron, behavior, synapse, astrocytic Ca^2+^ signaling

## Abstract

Behavior arises from coordinated brain-wide neural and glial networks, enabling organisms to perceive, interpret, and respond to stimuli. Astrocytes play an important role in shaping behavioral output, yet the underlying molecular mechanisms are not fully understood. Astrocytes respond to intrinsic and extrinsic cues with calcium (Ca^2+^) fluctuations, which are highly heterogeneous across spatio-temporal scales, contexts, and brain regions. This heterogeneity allows astrocytes to exert dynamic regulatory effects on neuronal function but has made it challenging to understand the precise mechanisms and pathways linking astrocytic Ca^2+^ to specific behavioral outcomes, and the functional relevance of these signals remains unclear. Here, we review recent literature uncovering roles for astrocytic Ca^2+^ signaling in a wide array of behaviors, including cognitive, homeostatic, and affective focusing on its physiological roles, and potential pathological implications. We specifically highlight how different types of astrocytic Ca^2+^ signals are linked to distinct behavioral outcomes and discuss limitations and unanswered questions that remain to be addressed.

## Graphical Abstract

Astrocytic Ca^2+^ regulates behavioral output. Confocal images of astrocyte network (top left) and single astrocyte (top right) from P14 mouse visual cortex expressing AAV5 GfaABC1D lck-eGFP, scale bar = 10 μm. Diagram (bottom) outlining differential behavioral outputs and corresponding changes in calcium levels. *denotes conditions where different effects were reported depending on brain region. 
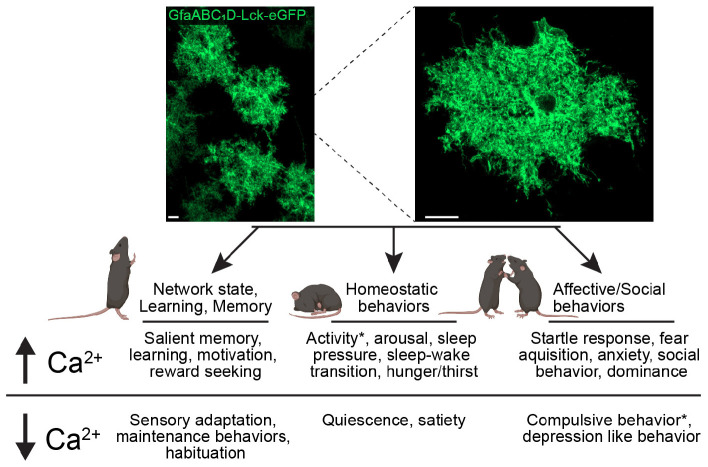

## Introduction

Behavior emerges from the coordinated activity of brain-wide cellular networks including neurons and glia, which modulate an organism’s ability to perceive, interpret, and appropriately respond to environmental or intrinsic stimuli (McCormick et al., 2020; Wu Y. et al., 2024). It is now well established that astrocytes, a major type of glia, play key roles in shaping behavioral responses by regulating multiple aspects of neuronal function such as synaptic formation and function (Farhy-Tselnicker and Allen, 2018; Tan et al., 2021), plasticity (Ota et al., 2013; Wang et al., 2022), and circuit dynamics (Hirrlinger and Nimmerjahn, 2022; Oliveira and Araque, 2022). Tiling every region of the brain where they closely associate with the vasculature as well as hundreds of thousands of synapses in rodents (Bushong et al., 2002; Sofroniew, 2021; Hösli et al., 2022; Lorin et al., 2024) [and millions in humans (Oberheim et al., 2009; Oberheim et al., 2012)], astrocytes monitor the brain’s microenvironment and tune the responses of neurons and other glial cells to network activity and metabolic states (Jha et al., 2019; Nutma et al., 2020; Rueda-Carrasco et al., 2021; Xie et al., 2022; Molina-Gonzalez et al., 2023; Hu et al., 2024; Imrie et al., 2024). However, the specific cellular mechanisms linking astrocytic function and behavioral outputs are not fully understood.

Astrocytic signaling is primarily mediated by changes in calcium (Ca^2+^) levels [reviewed in Khakh and Deneen (2019), Goenaga et al. (2023), Ahrens et al. (2024), and Bai et al. (2024)] which are highly heterogeneous across multiple spatial and temporal scales (Srinivasan et al., 2015; Bindocci et al., 2017; Semyanov, 2019).

**FIGURE 1:**
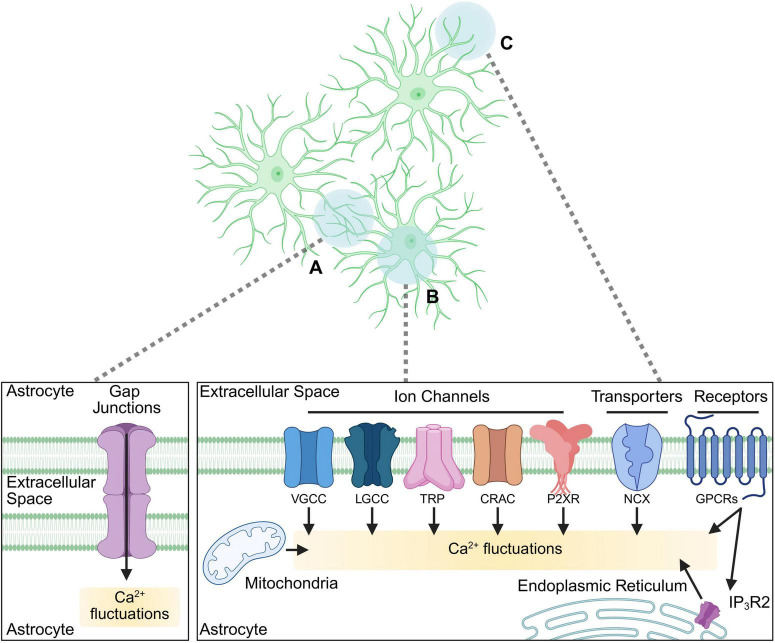
Cellular pathways involved in astrocytic Ca^2+^ signaling. A diagram of astrocytic cellular compartments (top panel) and the signaling pathways involved in Ca^2+^ dynamics (bottom panels) within them. (A) Astrocytic Ca^2+^ signals via gap junctions facilitate astrocytic network communication. (B, C) Diverse pathways mediated via receptors, transporters, channels, and cellular organelles activating Ca^2+^ fluctuations in soma and major processes (B), as well as in fine processes (C) such as those contacting synapses (synapses not depicted). VGCC, voltage-gated Ca^2+^ channels; LGCC, ligand-gated Ca^2+^ channels (including ionotropic receptors such as NMDAR); TRP, transient receptor potential channel; CRAC, Ca^2+^ release-activated Ca^2+^ channel; P2XR, purinergic receptor P2X; NCX, Na^+^-Ca^2+^ exchanger; GPCR, G protein coupled receptor; IP3R2, inositol trisphosphate receptor type 2. Biorender.

Microdomain Ca^2+^ transients occur within the fine astrocytic processes that contact synapses, allowing modulation of synaptic activity by influencing gliotransmitter release, neurotransmitter uptake and extracellular ion homeostasis with precise spatio-temporal control (Shigetomi et al., 2010; Shigetomi et al., 2013a; Agarwal et al., 2017; Ahmadpour et al., 2021; Lia et al., 2021; Denizot et al., 2022) (Figure 1). Astrocytes can also generate larger-scale somatic Ca^2+^ changes which are primarily mediated by the release of Ca^2+^ stored in the endoplasmic reticulum (ER) (Srinivasan et al., 2015; Stobart et al., 2018; Sherwood et al., 2021). These fluctuations can occur spontaneously as well as through extrinsic signaling via G protein coupled receptor (GPCR)-mediated inositol trisphosphate (IP3) pathway, which in astrocytes is predominantly mediated via the inositol trisphosphate 3 receptor type 2 (IP3R2). It was shown that unlike in neurons, activating both modulatory (Gα_q_) and inhibitory (Gα_i_)-coupled GPCRs in astrocytes can elicit stored Ca^2+^ release, demonstrating the complex nature of astrocytic Ca^2+^ dynamics (Kofuji and Araque, 2021b; Vaidyanathan et al., 2021; Denizot et al., 2022). Somatic Ca^2+^ fluctuations can further propagate as intracellular Ca^2+^ “waves” or “surges,” which can travel within the cell body and processes and extend to other astrocytes via gap junctions, thus facilitating communication within glial networks and coordinating activity across brain regions (Scemes and Giaume, 2006; Fujii et al., 2017).

The sources of astrocytic cytosolic Ca^2+^ depend on the type of signaling initiated in the cell (Figure 1). While the ER serves as a primary reservoir for somatic fluctuations (Okubo, 2020) in response to GPCR activation, it is also shown to contribute to microdomain signals (Lia et al., 2021; Denizot et al., 2022). Further, the mitochondria, which interact with the ER, play a dual role by both buffering intracellular Ca^2+^ levels and regulating its release, thereby shaping the amplitude and duration of Ca^2+^ transients (Agarwal et al., 2017; MacVicar and Ko, 2017; Serrat et al., 2022). Extracellular Ca^2+^ influx also contributes significantly to astrocytic activity, occurring through multiple pathways including transient receptor potential (TRP) channels (Shigetomi et al., 2013b; Verkhratsky et al., 2014; Bosson et al., 2017), voltage-gated Ca^2+^ channels (VGCCs) (Cheli et al., 2016; Zamora et al., 2020), purinergic P2X receptors (Ahmadpour et al., 2021), transporters (such as Na^+^-Ca^2+^ exchanger, NCX) (Rose et al., 2020), store-operated Ca^2+^ entry (SOCE) mechanisms (Toth et al., 2019) (such as Ca^2+^ Release-Activated Ca^2+^ (CRAC) channels), and activation of Ca^2+^ permeable ionotropic receptors (or ligand-gated channels, LGCCs), such as N-methyl-D-aspartate receptors (NMDARs) which, in addition to neurons, are also expressed by astrocytes [reviewed in Imrie et al. (2024)]. How these signals intersect to produce cellular responses is not well understood, but the dynamic relationship between these sources and the cellular pathways they activate allows astrocytes to integrate diverse signals and regulate neuronal activity with high versatility.

The development of tools allowing for the visualization, quantification, and manipulation of astrocytic Ca^2+^ signals has been central to understanding their physiological relevance. Visualization of astrocytic Ca^2+^ signals is largely executed through imaging of fluorescent dyes or genetically encoded Ca^2+^ indicators (GECIs) such as green or red fluorescent protein conjugated calmodulin-M13 peptides (G/RCaMP) (Lohr et al., 2021). These indicators are used across multiple experimental models including cultured cells (Ryu et al., 2024), *ex vivo* slices (Srinivasan et al., 2015), or *in vivo* in head-fixed or freely behaving animals (Qin et al., 2020; Gau et al., 2024). Additionally, astrocytic Ca^2+^ signals can be manipulated to assess upstream effectors and downstream consequences. These include reductions via chelators (Sasaki et al., 2014), genetic removal of IP3R2 (Srinivasan et al., 2015), or via Ca^2+^ extrusion pumps such as CalEx (Yu et al., 2018), or activation through stimulation of GPCRs (Kofuji and Araque, 2021b), including chemogenetic stimulation of designer receptors exclusively activated by designer drugs (DREADDS) (Roth, 2016; Lee et al., 2023). Several analysis tools to decode astrocytic Ca^2+^ signals have been utilized including GECIquant (Sharmila, 2019) and Astrocyte Quantitative Analysis (AQuA) (Wang et al., 2019), providing high resolution signal detection and quantification. While these methods provide robust representations of Ca^2+^ events, their physiological relevance should be carefully considered, as Ca^2+^ buffering by indicators, insufficient labeling (such as lack of signal in the fine processes), or off target effects (such as gliosis due to overexpression of modified fluorescent proteins) are a possibility (Semyanov et al., 2020). Furthermore, when manipulating astrocytic Ca^2+^ signaling, discerning whether the effects accurately represent endogenous physiological activity is a challenge. For example, recent work using *in vivo* imaging of mouse cortex has shown that activation of G_q_-DREADDs strongly yet transiently increases astrocytic Ca^2+^, followed by persistent suppression of Ca^2+^ signals (Vaidyanathan et al., 2021). For detailed reviews on this topic see: (Khakh and McCarthy, 2015; Shigetomi et al., 2016; Semyanov et al., 2020; Bai et al., 2024).

A large body of work in recent years using primarily mammalian models has strongly implicated astrocytic Ca^2+^ signaling in a wide range of central nervous system functions including behavioral output [for further reading see (Guerra-Gomes et al., 2018; Kofuji and Araque, 2021a; Lyon and Allen, 2022)], while disruptions in astrocytic Ca^2+^ homeostasis have been observed in neurological and psychiatric disorders in both rodent models and human tissue (Shah et al., 2022; Sobolczyk and Boczek, 2022; González-Arias et al., 2023), underscoring the importance of understanding these processes in both physiological and pathological contexts. Despite these prominent findings, the functional relevance of astrocytic Ca^2+^ signaling has been a controversial topic (Nizar et al., 2013; Takata et al., 2013; Bonder and McCarthy, 2014; Jego et al., 2014; Petravicz et al., 2014), and a comprehensive understanding of the mechanisms by which astrocytic Ca^2+^ signaling modulates behavior is lacking. In this review, we highlight recent advances in our understanding of how astrocytic Ca^2+^ signaling contributes to behavioral output focusing on findings which characterize astrocytic Ca^2+^ dynamics underlying various cognitive and emotional processes and neural circuit function. By integrating results from cellular, systems, and behavioral studies, we provide a comprehensive perspective on the role of astrocytic Ca^2+^ signaling in brain function and subsequent behavioral responses.

## Astrocytic Ca^2+^ signaling regulates cortical network states, learning, and memory

### Cortical network states

Cortical network states define how populations of brain cells interact and process information (Buonomano and Maass, 2009; Wu M. et al., 2024). In this context, “states” refers to distinct activity patterns which facilitate different kinds of behaviors and can be measured by recording local field potentials to quantify oscillatory dynamics (Colgin, 2011; Liu et al., 2022; Chen et al., 2024). For instance, deep sleep is characterized by delta oscillations at 0.5–4 Hz (Kim et al., 2019), while sensory processing, attention, and working memory are characterized by gamma oscillations at 30–100 Hz (Buzsáki and Wang, 2012). Astrocytic Ca^2+^ signaling diversely regulates cortical state maintenance and transitions, underlying multiple modes of behavioral output. Studies pairing *in vivo* Ca^2+^ imaging using genetically encoded Ca^2+^ indicators (GECIs) with behavioral tracking or electrophysiology show that cortical astrocyte Ca^2+^ fluctuations are critical for cortical network state switching, which drives initiation and cessation of behaviors including sleep, arousal, feeding, and exploration (Poskanzer and Yuste, 2016; Reitman et al., 2023; Gau et al., 2024). It was shown that Ca^2+^ signals within the fine astrocytic processes underlie a switch to a slow-oscillation dominated state which is critical for behavioral regulation in both quiescent and active states (Anand et al., 2024), and which is associated with enhanced extracellular glutamate levels (Poskanzer and Yuste, 2016). Interestingly, astrocytic Ca^2+^ signals appear to be suppressed during habitual or familiar behaviors, but increase during unexpected behaviors, such as exploration of novel stimuli. Notably, these astrocytic Ca^2+^ responses decline with repeated exposure, suggesting an adaptive mechanism for encoding contextual behavioral salience changes (Gau et al., 2024).

State dependent cortical astrocyte Ca^2+^ signals are encoded by the distinct actions of specific neurotransmitters and neuromodulators. Studies leveraging *in vivo* astrocytic Ca^2+^ imaging in combination with neurotransmitter uncaging show that brief neurotransmitter input leads to long lasting network-wide astrocyte Ca^2+^ changes, which may serve as a mechanism for prolonged neuronal network activity integration. Glutamate and GABA uncaging both induced prolonged and spatially extensive astrocyte Ca^2+^ activity, with glutamate preferentially increasing propagative Ca^2+^ waves, which appear to modulate information flow within astrocytic networks (Cahill et al., 2024). Importantly, in these studies, astrocytic Ca^2+^ responses were context dependent, with baseline propagative activity inversely correlated with responsiveness to neurotransmitter input. Additionally, norepinephrine (NE) is shown to drive astrocyte Ca^2+^ transients involved in cortical synchronization, important for transitions from quiescent to active states and behavioral timing (Reitman et al., 2023; Gau et al., 2024), while acetylcholine (ACh) modulates cortical astrocyte Ca^2+^ transient amplitude during novel experience (Gau et al., 2024).

### Sensory perception

Network states allow organisms to make sense of environmental stimuli in a contextually relevant manner, supporting a role for astrocytic Ca^2+^ activity in sensory perception and processing. Indeed, recent work using Ca^2+^ imaging and electrocorticography (ECoG) in mice shows that astrocytes in the primary somatosensory cortex exhibit stimulus dependent Ca^2+^ elevations in response to sensory stimulation, which temporally correlate with neural network activity, specifically gamma oscillations, linked to sensory processing and cortical excitability. In mice lacking the ER receptor IP3R2 and subsequent store-released Ca^2+^ signaling in astrocytes, gamma oscillation steady state is increased and its temporal decline during sensory stimulation is diminished. Further, manipulation of astrocytic Ca^2+^ levels using a chemogenetic approach with Gα_q_-coupled designer receptors exclusively activated by designer drugs (G_q_-DREADDs) reduced cortical gamma frequency responses to sensory stimulation, suggesting that astrocytic Ca^2+^ plays an important role in modulating sensory-evoked gamma activity by regulating its upper limits (Lines et al., 2020). Notably, since DREADD mediated activation of astrocytic Gα_q_-coupled pathways is pancellular, it may mask the contributions of compartmentalized astrocytic Ca^2+^ signals in this context. Astrocytic microdomain Ca^2+^ transients are also implied in cortical responses to sensory stimulation. Virally mediated knock-down of astrocytic NMDARs, activation of VGCCs, and metabotropic signaling caused neural network desynchronization and impaired adaptation to whisker stimulation in the mouse barrel cortex (Ahmadpour et al., 2024).

### Learning

Dynamic modulation of neural responses to relevant stimuli underlies the acquisition of behavior, which can be innate or learned (Shahaf and Marom, 2001; Aizenberg et al., 2015; Inácio et al., 2025). Encoding of reward drives learning through changes in synaptic plasticity brought about by signaling molecules that transmit information regarding expected and elicited outcomes (Ding et al., 2022). Recent evidence provides a framework for the role of astrocytic Ca^2+^ signaling in these processes. Using the AstroLight tool, which employs a light-sensitive transcriptional switch that only activates gene expression in the presence of high intracellular Ca^2+^ and blue light application, it was shown that astrocytes in the nucleus accumbens (NAc) form ensembles that mediate cue-motivated behaviors in mice. Though AstroLight is a powerful tool for identifying contextually respondent astrocytes, the manipulation of broad populations of astrocytes using opsins may lead to cellular activity changes that deviate from physiological norms. Fiber photometry and Ca^2+^ imaging showed progressive recruitment of astrocytic Ca^2+^ activity in the NAc during cue–reward learning. Optogenetic and chemogenetic modulation of these ensembles is sufficient to modulate behavior, demonstrating that astrocytes integrate motivational information through Ca^2+^ signaling to contribute to decision making processes (Serra et al., 2025). These findings are consistent with evidence in the globus pallidus externus, where astrocytic Ca^2+^ is shown to gradually reduce as habit formation progresses (Kang et al., 2023), while in the hippocampus, CA1 astrocytes chronically imaged *in vivo* were shown to gradually “ramp up” Ca^2+^ activity during reward seeking in a previously learned location (Doron et al., 2022). In the NAc these effects are diversely mediated by unique glutamatergic circuits, displaying input region-specific astrocytic Ca^2+^ responses: inputs from the medial prefrontal cortex (mPFC) trigger high levels of Ca^2+^ activity in astrocytes of the NAc core and shell, amygdala inputs enhance astrocytic Ca^2+^ signals in the dorsal NAc, while ventral hippocampus inputs broadly activate astrocytic networks (Serra et al., 2022).

### Memory

Memory is essential for learning, and the neural and glial mechanisms underlying these processes are closely interrelated, mutually driving behavioral output shaped by experience. Accumulated evidence suggests an important role for astrocytic Ca^2+^ in multiple aspects of memory function including formation, consolidation, and retrieval (Huang et al., 2020; Escalada et al., 2024). *In vivo* Ca^2+^ imaging studies in mice show that hippocampal CA1 astrocytes integrate information from salient past events such that Ca^2+^ signals from distal astrocytic processes are followed by Ca^2+^ changes in the soma, generating specific patterns of networked Ca^2+^ activity dependent on arousal state and past Ca^2+^ signaling events (Rupprecht et al., 2024). This also has important implications for neuronal plasticity necessary for memory formation and allocation, which are enhanced by optogenetic or chemogenetic G_q_-DREADD induced astrocytic Ca^2+^ changes which facilitate NMDAR-dependent long-term potentiation in CA1 (Adamsky et al., 2018; Suthard et al., 2023a). These effects may in part be mediated by the activity of astrocytic α4-nAChRs, which drive Ca^2+^ transients that regulate the co-agonist supply for NMDARs, strengthening temporal association memory, an effect that was diminished by attenuation of astrocytic Ca^2+^ (Ma et al., 2023). Recent work also reveals an important role for Gα_*i*_-GPCR-mediated astrocytic Ca^2+^ changes in CA1, which impair remote but not recent memory when chemogenetically modulated during learning. This manipulation also affected neuronal activity in the anterior cingulate cortex through the disruption of CA3 to CA1 communication, indicating an astrocytic role in circuit-specific regulation of memory (Kol et al., 2020).

This was also observed in the basolateral amygdala, where astrocytic Ca^2+^ extrusion by virally mediated expression of the CalEx pump impaired context dependent memory recall (Sun et al., 2024). The molecular mechanisms underlying astrocytic Ca^2+^ signaling in memory are still largely unknown, but work leveraging electrophysiological recording in mouse brain slices has uncovered that store-operated Ca^2+^ release-activated Ca^2+^ (CRAC) channels comprised of Orai1 and STIM1 are necessary for the development of sustained and oscillatory Ca^2+^ signals in response to GPCR stimulation, and subsequent release of ATP in CA1 (Toth et al., 2019). Additionally, rescuing STIM1 expression enhanced long-term plasticity in Alzheimer’s disease (AD) models in female mice, which display decreased astrocytic Ca^2+^ activity associated with store-released Ca^2+^ dysfunction (Lia et al., 2023).

To summarize, astrocytic Ca^2+^ signaling is emerging as a central regulator in cortical network state maintenance, sensory perception, learning, and memory, which are all critical components in the acquisition and elicitation of behavioral output. Through neurotransmitter-specific responses and regionally distinct signaling mechanisms, astrocytes adaptively encode environmental stimuli and behavioral salience via dynamic changes in Ca^2+^ fluctuations, reinforcing their importance in experience-dependent plasticity. Though recent work has made great progress in identifying the specific effects of upregulation or abrogation of astrocytic Ca^2+^ signaling in these functions, studies investigating the specific molecular mechanisms and subcellular pathways that are activated in response to astrocytic Ca^2+^ manipulation are lacking. Expanded investigations focusing on the interactions between CRAC mediated Ca^2+^ entry and other Ca^2+^ sources *in vivo* will be important for identifying how these pathways contribute to behavior. Ultimately, studies describing the context, temporal, and circuit dependent mechanisms by which astrocytic Ca^2+^ signaling mediates cortical state, learning, and memory will be critical for determining how astrocytes regulate behavioral output at its earliest stages.

## Astrocyte Ca^2+^ signaling regulates homeostatic behaviors

Homeostatic behaviors such as sleep-wake cycles and food intake are essential for survival, allowing organisms to maintain stable internal conditions despite changes in their environment. These behaviors originate when conditions deviating from physiological ranges are detected and integrated by both neuronal and glial networks to generate appropriate responses (Simard and Nedergaard, 2004; Lam, 2010; Rosenberg and Rao, 2021; Ahn et al., 2022). Through their extensive interactions with neuronal synapses and CNS vasculature, astrocytic Ca^2+^ changes tune these behavioral outputs in a contextually relevant manner (Parpura and Verkhratsky, 2012; Murphy-Royal et al., 2017; Lee et al., 2021; Tewari et al., 2024).

### Circadian rhythmicity

Circadian rhythmicity is fundamental to the maintenance of homeostatic functions in most animals (de Assis and Oster, 2021; Mortimer et al., 2025). Astrocytes express genes encoding the molecular clock and show robust circadian rhythmicity (Barca Mayo et al., 2019; Womac et al., 2009; Brancaccio et al., 2017; Ruben and Hogenesch, 2017; Whalley, 2017; McCauley et al., 2020; Coomans et al., 2021; Hastings et al., 2023; Ryu et al., 2024). In mammals, core circadian output is generated within the suprachiasmatic nucleus (SCN) of the hypothalamus, where astrocytic Ca^2+^ activity is anti-phase to neuronal Ca^2+^ activity, as shown by long term imaging of *ex vivo* organotypic mouse brain slices expressing virally delivered GECIs. This anti-phasic oscillatory pattern was observed in both the soma and microdomains, with the latter showing particularly robust signals in the dorsal SCN, implying that Ca^2+^ activity in astrocytic processes has important functional relevance in this region. Indeed, this study identified that the enhanced astrocytic Ca^2+^ signaling corresponds with astrocytic glutamate release which suppresses neural activity though increased GABAergic tone, mediated by astrocytic NMDARs in the dorsal SCN (Brancaccio et al., 2017).

Astrocytic Ca^2+^ activity also shows circadian rhythmicity *in vivo*. Leveraging long range fiberscope imaging in behaving mice, it was shown that cortical astrocytes exhibit robust somatic Ca^2+^ fluctuations corresponding with the animal’s activity, with higher frequency and amplitude during active periods than during quiescence (Gau et al., 2024). These oscillations in somatic Ca^2+^ signals may be driven by circadian changes in expression of the glial ER receptor IP3R2. Recent evidence in primary cultured cortical astrocytes demonstrates that rhythmic expression of heat shock factor-1 regulated protein (HERP) regulates the degradation of IP3Rs in a circadian manner. IP3R2 expression was found to be anti-phase to HERP expression, and ATP induced somatic Ca^2+^ transients, which are normally higher during subjective night (corresponding to active periods for rodent astrocytes), lost rhythmicity in Herp knockdown astrocytes (Ryu et al., 2024). HERP mediated IP3R2 Ca^2+^ signaling was also linked to the rhythmic phosphorylation of connexin 43, which is shown to reduce gap junction conductance (Solan and Lampe, 2014; Nimlamool et al., 2015), thereby modulating Ca^2+^ signaling across astrocyte networks.

### Sleep/wake

During sleep/wake cycles, astrocytic Ca^2+^ signaling is heterogeneous across brain regions. Outside of the SCN, astrocytic Ca^2+^ fluctuations generally correlate with activity levels, decreasing/desynchronizing during sleep and amplifying/synchronizing during wakefulness (Bojarskaite et al., 2020; Ingiosi et al., 2020; Vaidyanathan et al., 2021; Peng et al., 2023; Gau et al., 2024; Péter and Héja, 2024; Ryu et al., 2024). Conversely, some brain regions such as the basolateral forebrain (BF) and brainstem exhibit increased astrocytic Ca^2+^ signaling during rapid eye movement (REM) sleep, characterized by high levels of neural activity, muscle atonia, and dreaming. Chemogenetic modulation of astrocytic Ca^2+^ using G_q_-DREADDs generally reduced REM sleep, while differentially impacting brain activity in the delta frequency associated with non-REM sleep, reducing it in the BF and increasing it in the brainstem, suggesting that astrocytic Ca^2+^ dependent modulation of sleep/wake activity is both sleep state and brain region specific (Peng et al., 2023).

Recent work demonstrates that the arousal inducing effects of astrocytic Ca^2+^ are prominent at the network level, with global increases in intracellular astrocytic Ca^2+^ waves underlying the transitions from quiescent to active behavior, an effect which was strongly suppressed by inhibition of NE release from presynaptic terminals, suggesting an important role for this neuromodulator in elevating astrocytic Ca^2+^ during arousal (Gau et al., 2024). Consistently, live imaging of GECI expressing astrocytes shows that in the barrel cortex (BC), NE release from the locus coeruleus toggles a switch from small Ca^2+^ signals (observed in the quiescent BC during whisker stimulation) to large Ca^2+^ waves (observed in the awake BC during whisker stimulation) in the astrocytic processes (Wang et al., 2023). Interestingly, astrocytes in the BC also show large Ca^2+^ fluctuations in somata and processes underlying slow wave sleep to arousal, but not REM to arousal transitions (Bojarskaite et al., 2020). Combining Ca^2+^ imaging with local field potential recording in the BC showed that the small Ca^2+^ transients (characteristic of BC astrocytes during sleep) reduced EPSP amplitude, suppressing sensory transmission (Wang et al., 2023) and providing a potential mechanism for the role of astrocytic Ca^2+^ signaling in sleep modulation in this region. Duality in astrocytic Ca^2+^ signals is also observed in *drosophila*, where Ca^2+^ increases in somas and processes mediated by the astrocyte specific temperature sensitive cation channel dTrpA1 resulted in two unique phenotypes: a fast elevation in sleep which occurred at night, and delayed but persistent increase in sleep during the day supporting that in flies, astrocytic Ca^2+^ signaling encodes sleep pressure (Blum et al., 2021; Srinivasan, 2021). Taken together these findings suggest that astrocytic Ca^2+^ fluctuations are multimodal and intimately involved with state transitions between sleep and arousal.

Astrocytic Ca^2+^ activity is also implied in sleep architecture, which refers to the structured organization and progression of sleep stages across a sleep period (Younes et al., 2022). In studies combining Ca^2+^ imaging in behaving, head-fixed mice with electrocorticography (ECoG), it was shown that inhibition of ER released astrocytic Ca^2+^ through deletion of IP3R2 causes slow wave sleep to become fragmented, corresponding with reduced ECoG delta power (Bojarskaite et al., 2020), in agreement with studies showing that high frequency somatic astrocytic Ca^2 +^ oscillations in the delta (and theta) frequency are critical for modulating slow wave sleep (Péter and Héja, 2024). The sleep phenotypes resulting from IP3R2 KO are likely linked to an inability to respond to both Gα_q_ and Gα_*i*_-coupled GPCR-mediated signaling pathways, which are shown to regulate sleep duration and depth, respectively (Vaidyanathan et al., 2021).

### Nutrient intake

Additional homeostatic behaviors modulated by astrocytic Ca^2+^ dynamics include feeding and drinking. During these behaviors under *ad libitum* conditions, cortical astrocytic Ca^2+^ transients are suppressed (Gau et al., 2024). However, after starvation or water restriction, astrocytic Ca^2+^ signals increase in response to food or water, and to a greater extent when visual or olfactory cues are presented to deprived mice while food is inaccessible. These data suggest that cortical astrocyte activity drives these behaviors and is flexible to neuromodulation dependent on internal motivation state (Gau et al., 2024). Indeed, in the murine arcuate nucleus (ARC) of the hypothalamus, which plays a central role in feeding behaviors (Zhang et al., 2019), chemogenetic G_q_-DREADD manipulation of astrocytes induced robust somatic Ca^2+^ signaling driving food intake through increased activation of orexigenic AgRP/NPY neurons, which inhibit satiety promoting neurons (Chen et al., 2016; Zhang et al., 2019). These findings correspond with reports showing that astrocytes modulate feeding behavior through the regulation of extracellular adenosine levels, which is coupled to astrocytic intracellular Ca^2+^ levels (Yang et al., 2015). Further, hypothalamic astrocytes downstream of ARC nucleus show robust leptin receptor expression which induces somatic Ca^2+^ signals upon stimulation in mice. Leptin, a hormone produced by adipocytes, has a prominent role in satiety behavior, with increased levels leading to leptin resistance in obesity and metabolic syndromes (Engin, 2017; Balland et al., 2019; Zhang et al., 2019). Astrocytic leptin receptors and the related Ca^2+^ transients are increased in mice subject to diet induced obesity (DIO), suggesting a role for astrocytic Ca^2+^ in obesity linked metabolic disruption (Hsuchou et al., 2009). Accordingly, a recent report demonstrates that DIO in mice increases the frequency and amplitude of Ca^2+^ signals in astrocytes in the paraventricular nucleus (PVN), ARC, and dorsomedial nucleus of the hypothalamus (DMH), without affecting astrocytic Ca^2+^ mobilization in the ventromedial nucleus of the hypothalamus (VMH). Additionally, chemogenetic manipulation of these Ca^2+^ signals had aggravating (via G_q_-DREADD) or alleviating (via G_*i*_-DREADD) effects on metabolic condition in mice subject to DIO (Herrera Moro Chao et al., 2022).

Taken together, these findings provide critical insight into the role of astrocytic Ca^2+^ signaling in the modulation of homeostatic behaviors. Astrocytes regulate these processes through Ca^2+^ fluctuations at the network level and in multiple subcellular compartments in a heterogeneous manner dependent on the brain region and physiological context. Given its central role in arousal, particularly in sleep-wake transitions characterized by enhanced astrocytic Ca^2+^ signaling, NE is emerging as a key component in these processes, potentially mediated by astrocytic α1-adrenergic receptors. However, the specific interactions between neuronal NE release and astrocytic Ca^2+^ dynamics remain unclear, calling for further targeted investigations, especially with respect to spatially and functionally distinct astrocytic Ca^2+^ signals across brain regions. Furthermore, very little is known about the roles of astrocytic Ca^2+^ in other survival behaviors such as defensive responses, or how astrocytic Ca^2+^ integrates with metabolic signals, such as leptin and adenosine. Uncovering these roles may provide insights into how disruptions in relevant pathways contribute to disorders of sleep, metabolism, and circadian misalignment.

## Astrocytic Ca^2+^ signaling regulates affective and social behaviors

### Fear

Affective behaviors such as fear and anxiety, are fundamental to organismal responses to environmental stimuli (Raber et al., 2019; Mendl and Paul, 2020), and astrocytic Ca^2+^ signaling has been widely identified as a major component in mediating these behaviors. *In vivo* Ca^2+^ imaging during air-puff evoked startle demonstrated robust, global astrocytic Ca^2+^ responses in the cortex which consisted of a fast α1-adrenoceptor dependent spike in somatic Ca^2+^, and a phasic Ca^2+^ response with both early and late components within the astrocytic processes that was unaffected by α1-adrenoceptor blockade. In IP3R2 KO mice, somatic signals and early responses within the astrocytic processes were abrogated, but the late component was still readily identifiable, underscoring the complexity of compartmentalized astrocyte activation in response to relevant stimuli (Srinivasan et al., 2015). This air-puff-evoked startle response could be attenuated through expression of the GPCR signaling inhibitor, iβARK (Nagai et al., 2021). Astrocytic Ca^2+^ transients also mediate startle responses in zebrafish, with Ca^2+^ propagating bidirectionally from astrocytes in the rostral spinal cord through gap junctions in glial networks in a glutamate dependent manner, requiring adrenergic signaling for propagation in the hindbrain (Orts-Del’Immagine et al., 2022).

Neural mechanisms underlying startle responses are tightly interrelated with processes governing the more complex acquisition of fear, which engages numerous brain regions responsible for threat detection, stimulus integration, and contextual memory (de Haan et al., 2018; Zheng and Schmid, 2023). Recent work implies a prominent role for astrocytic Ca^2+^ signals in the modulation of these effects in the basolateral amygdala (BLA). During foot shock, BLA astrocytic Ca^2+^ signals were significantly elevated relative to non-shocked controls, indicating their role in encoding stimulus salience. Fascinatingly, different stages of fear acquisition appear to engage potentially distinct populations of astrocytes with unique Ca^2+^ kinetics (Suthard et al., 2023b). In the medial subdivision of the central amygdala (CeM), astrocytes respond to both endogenous endocannabinoid or exogenous G_q_-DREADDs stimulation with robust Ca^2+^ transients, which enhanced inhibitory signaling at lateral central amygdala to CeM synapses, dampening excitatory signaling at BLA-CeM synapses and reducing fear expression in a delayed fear conditioning paradigm (Martin-Fernandez et al., 2017). Together these results demonstrate that context, population, and synapse specific astrocytic Ca^2+^ signals are highly diverse across different components of fear behavior.

### Anxiety

While startle and fear represent acute responses to aversive stimuli, anxiety is characterized by a prolonged anticipation of and often disproportionate response to potential danger (Duval et al., 2015). Extruding Ca^2+^ from NaC astrocytes by overexpression of the CalEx pump led to pronounced reductions in anxiety behavior, increasing exploratory behaviors in mice. However, this manipulation also increased compulsive behaviors, such as perseverative responses in five choice serial reaction time task and enhanced hedonia in sucrose preference test (Peyton et al., 2025). These findings underscore a potential role for astrocytic Ca^2+^ not only in anxiety, but at the intersection between anxiogenesis and compulsive hedonic behaviors, which is a major factor underlying addiction (Koob, 2008). The hippocampus is also strongly implicated in the development of anxiety like behaviors, and recent evidence demonstrates an important role for astrocytic Ca^2+^ signaling in these processes. *In vivo* Ca^2+^ imaging in head-fixed mice revealed robust increases in astrocytic Ca^2+^ during the anxiogenic phase of a virtual reality paradigm, while mice in non-anxiogenic phases had minimal Ca^2+^ elevations. Interestingly while most astrocytes within the field of view responded to the anxiogenic phase, a smaller fraction of astrocytes responded specifically to the non-anxiogenic phase, suggesting heterogeneously respondent hippocampal astrocyte populations, and reinforcing the notion that astrocytes encode behavioral salience (Cho et al., 2022). Others show that anxiety-linked astrocytic Ca^2+^ increases are specifically abundant in the ventral hippocampus during anxiogenic behaviors, and conditional knockout of astrocytic IP3R2 was anxiolytic, implying a role for store-released Ca^2+^ in anxiety modulation. In these studies, it was also shown that chemogenetic manipulation of astrocytic Ca^2+^ signaling increased anxiogenic conditions through the enhanced release of glutamate, which contributes to stress susceptibility through neuronal NMDAR stimulation which could be ameliorated with specific NMDAR antagonists (Li et al., 2024). These effects may be modulated by metabotropic glutamate receptor (mGluR) signaling. MGluR5 induces robust IP3 mediated Ca^2+^ release in astrocytes, and its specific knockdown is associated with reduced inhibitory synaptic inputs in CA1 which correspond with increased anxiety-like behaviors (Li et al., 2023). In mice with chronically activated G_q_-DREADDs, astrocytic Ca^2+^ signals in the ventral CA1 and anxiety-like behaviors were increased, but only in three month old mice, while six month old mice exhibited no change relative to controls (Suthard et al., 2023a). These findings highlight the importance of considering developmental timepoint, however, effects may also be due to compensatory astrocytic mechanisms, as chronic G_q_-DREADD stimulation would also generate chronic Ca^2+^ depletion.

### Depression

Persistent anxiety, among other factors, can contribute to the development of depression (Ross et al., 2017), an affective behavior characterized by prolonged emotional dysregulation and reduced motivation and pleasure. Given the findings linking aberrant astrocytic Ca^2+^ signaling to anxiety, it is unsurprising that it is also implied in depressive phenotypes. Reducing astrocytic Ca^2+^ via CalEx pump extrusion during a critical period in mouse development (postnatal week 2–3) led to synaptic hyperexcitation and depressive like behaviors in adults, including anti-social behaviors and prolonged immobility in tail suspension and forced swim tests which could be rescued through G_q_-DREADD stimulation (Luo et al., 2023). Further, in chronically corticosterone treated juvenile mice, mPFC astrocytic Ca^2+^ fluctuations were aberrant at baseline and reduced during social and exploration behaviors relative to untreated controls, and serotonin (5-HT) evoked astrocytic Ca^2+^ signals were diminished (González-Arias et al., 2023). These age specific findings introduce important questions regarding the developmental nature of astrocytic Ca^2+^ signals, and how disruptions at specific timepoints may contribute to long term consequences.

### Social behavior

Astrocytic Ca^2+^ signaling is also implicated in social behaviors, contributing to social interactions and disorders characterized by their dysregulation. Recent evidence shows that social dominance behaviors are modulated by these signals, with astrocytic Ca^2+^ increasing in the mPFC during assertive and resistant behaviors between male mice, with higher amplitude responses recorded in dominant mice compared to subordinates. The study also identified that these behaviors were mediated by astrocytic release of glutamate and ATP, which regulate cortical excitation/inhibition (E/I) balance (Noh et al., 2023). This in part may be modulated by store-released Ca^2+^ signaling, as IP3R2 KO mice exhibited a delay in the assertion of dominance behaviors relative to wild type controls, with no effect on competitive outcome, suggesting the involvement of other pathways in these behaviors (Guillot de Suduiraut et al., 2021). Moreover, astrocytic Ca^2+^ signaling is implied in the social behavioral deficits observed in autism spectrum disorders (ASD). Transplantation of ASD derived human astrocytes into mouse brains induced deficits including repetitive compulsive behaviors (perseverative digging) and attenuated fear memory. The ASD derived astrocytes elicited aberrant, exaggerated Ca^2+^ signals relative to wild type astrocytes, implying that increased Ca^2+^ and subsequent gliotransmitter release underlies ASD like behavioral dysfunction in mice (Allen et al., 2022). IP3R2 mediated Ca^2+^ transients are linked to ASD like behaviors in mice, with IP3R2 KO mice exhibiting antisocial behavior in a place preference test and increased repetitive behaviors including perseverative digging as well as compulsive-like grooming behaviors. These behavioral deficits, as well as GABAergic neurotransmission, which was abrogated by IP3R2 KO, could be rescued by treatment with ATP or ATPyS, a gliotransmitter which is reduced in IP3R2 KO mice, implying a potential mechanism for astrocytic store-released Ca^2+^ signals in ASD related behavioral pathology (Wang et al., 2021). In agreement with these results, repetitive grooming behaviors were also observed following CalEx pump-mediated extrusion of astrocytic Ca^2+^ which disrupted striatal microcircuits, suggesting the involvement of astrocytic Ca^2+^ in this brain region in ASD pathology (Yu et al., 2018).

Thus, astrocytic Ca^2+^ signaling plays a crucial role in modulating affective and social behaviors, influencing processes like startle response, fear acquisition, anxiety regulation, and social dominance. However, unanswered questions remain regarding specific facets of astrocytic Ca^2+^ in these processes, for instance: how does gap junction mediated networked Ca^2+^ activity contribute to the development and maintenance of fear linked behaviors? What are the long-term effects of Ca^2+^ dysregulation in affective disorders, and are they limited to specific subsets of astrocytes? As transplanted human ASD astrocytes induced pathological phenotypes in rodents, can this approach be leveraged to investigate other disorders? Future studies considering the heterogeneity of astrocytes that regulate these behaviors, such as regional subpopulations, molecular signatures, specific gliotransmitter release, and different Ca^2+^ signaling pathways will be imperative to the identification of precise regulatory mechanisms involved in these processes. Importantly, because many of the neural pathways involved and phenotypes observed overlap between affective and social behaviors, studies characterizing their outcomes should be attentive to how astrocytic Ca^2+^ dynamics differ across these distinct behavioral responses.

## Conclusion and perspectives

There is mounting evidence that astrocytic Ca^2+^ signaling is a prominent and fundamental regulator of neural processing, influencing a wide range of behaviors from sensory perception and learning to affective and social interactions. The dynamic and regionally heterogeneous nature of astrocytic Ca^2+^ fluctuations emphasize the importance of their role in the contextually specific encoding of critical determinants of behavior, including stimulus salience and transitions between activity states. While significant progress has been made in characterizing the diverse modes of Ca^2+^ activity correlating with behavioral outputs, questions remain regarding the molecular mechanisms underlying these effects as well as the relationships between astrocytic Ca^2+^ signaling and other neuromodulatory systems in the CNS that drive behavioral output. Investigating the role of neurotransmitters such as NE which is emerging as a major modulator of astrocytic Ca^2+^ activity, will be necessary to discern the distinct circuit dependent roles of astrocytic Ca^2+^ signaling in behavioral regulation. Additionally, attention to the different sources and types of Ca^2+^ signals, and the underlying mechanisms that induce or suppress them, will be imperative to understanding how astrocytes leverage Ca^2+^ to process information. Ascertaining which of these signals lead to gliotransmitter release, and in which contexts, will improve our understanding of how astrocytic Ca^2+^ modulates neuronal activity. Further, interactions with other glial cells in the behavioral context are largely unexplored. Future research leveraging high resolution imaging, genetic manipulations, and circuit level analyses will be critical to understanding the roles of astrocytic Ca^2+^ signals at the subcellular, single cell, and global network level. Such investigations will provide deeper insight into the role of astrocytic Ca^2+^ signaling in fundamental neurobiological processes involved in behavior and identify novel therapeutic targets for behavioral disorders.
